# Supplementary material for: Anti-Cancer Potential of Oxialis obtriangulata in Pancreatic Cancer Cell through Regulation of the ERK/Src/STAT3-Mediated Pathway
Source: Molecules. 2020 May 14;25(10):2301. doi: 10.3390/molecules25102301 (PMC7288118; doi:10.3390/molecules25102301)
Supplement: Supplementary file 1 [file molecules-25-02301-s001.pdf]

molecules
MDPI

Supplementary Materials

Anti-Cancer Potential of *Oxialis obtriangulata* in Pancreatic Cancer Cell through Regulation of the ERK/Src/STAT3-Mediated Pathway

Eun-Jin An [1,2], Yumi Kim [1,2], Seung-Hyeon Lee [1,2], Hyun Min Ko [1,2], Won-Seok Chung [1,3,*] and Hyeung-Jin Jang [1,2,4,*]

1 College of Korean Medicine, Kyung Hee University, 26, Kyungheedae-ro, Dongdaemun-gu, Seoul 02447, Korea; aej3866@naver.com (E.-J.A.); yumi0201@khu.ac.kr; (Y.K.); skyking27@naver.com (S.-H.L.); rhgusals93@naver.com (H.M.K.)
2 Department of Science in Korean Medicine, Graduate School, Kyung Hee University, Seoul 02447, Korea.
3 College of Korean Medicine, Kyung Hee University, 26, Kyungheedae-ro, Dongdaemun-gu, Seoul 02447, Korea
4 College of Korean Medicine and College of Pharmacy, Kyung Hee University, 26, Kyungheedae-ro, Dongdaemun-gu, Seoul, 02447, Korea
* Correspondence: omdluke@khu.ac.kr (W.-S.C.); hjjang@khu.ac.kr (H.-J.J.)

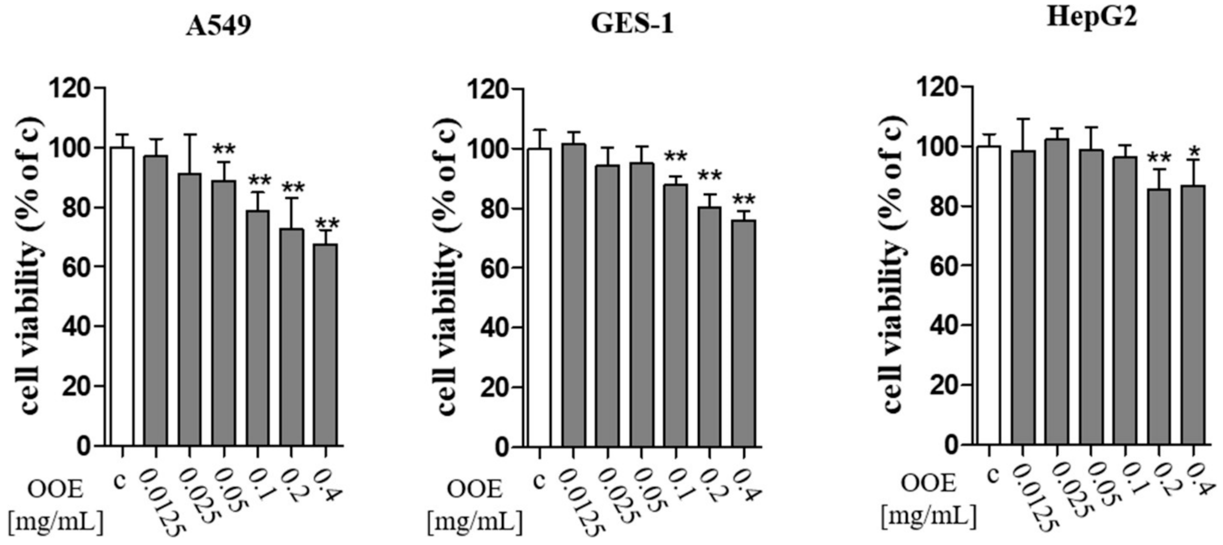

Figure S1. Effects of OOE on A549, GES-1 and HepG2 cells viability. Cells were seeded in 96-well plates and treated with OOE (0, 12.5, 25, 50, 100, 200, and 400 µg/mL) for 24 h. Cell viability was determined using MTT solution. The relative cell viability is shown as a bar graph compared with the control group (100%). MTT data are expressed as the mean ± S.D.

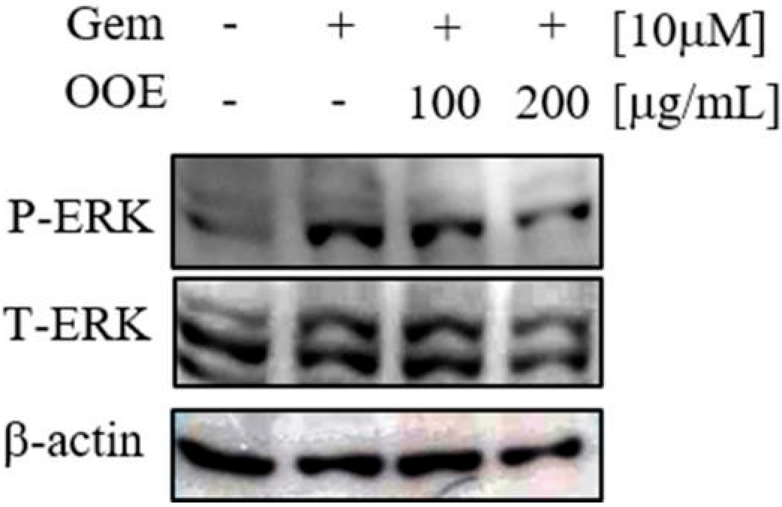

Figure 2. Effect of OOE on ERK expression which is overexpressed by Gemcitabine. Cells are seeded in 6 well plate with or without Gemcitabine(10μM) 24 h before treated with or without OOE for 3 h. pERK, TERK and actin were detected by western blot.

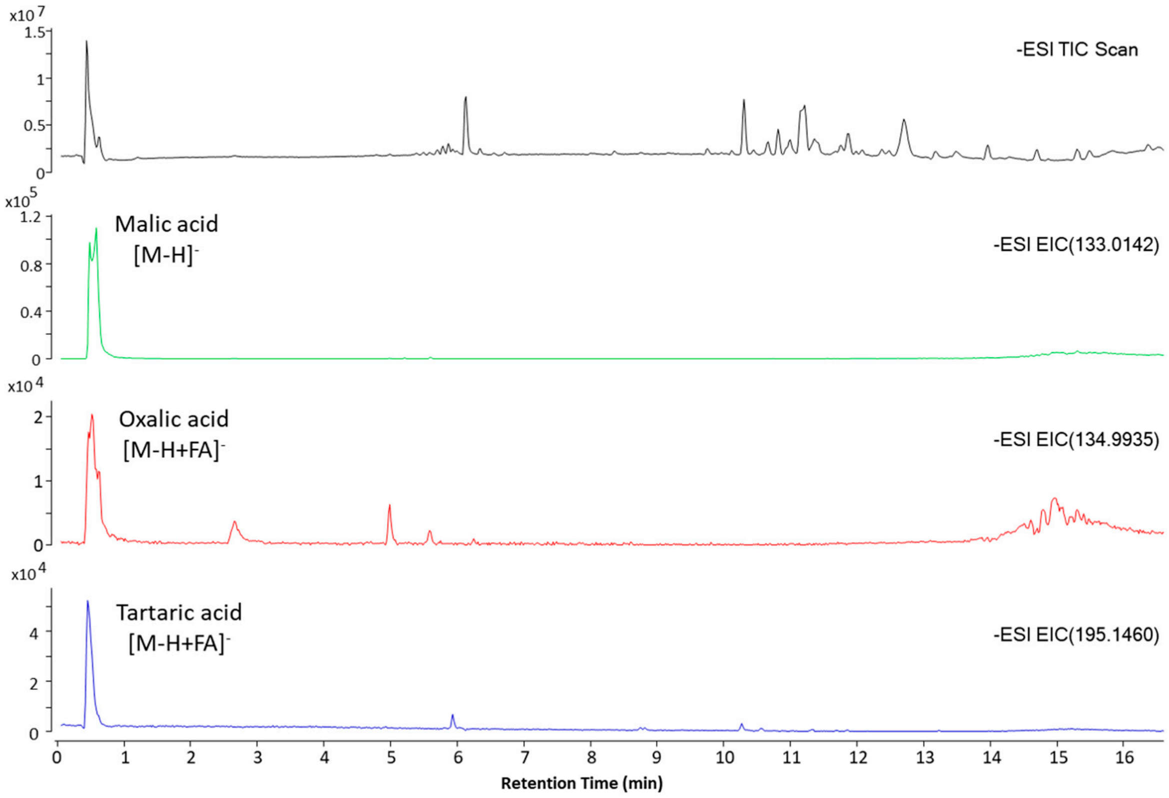

Figure S3. LC-MS analysis of OOE. LC-MS was performed to confirm the separation of components in OOE. A negative ion chromatogram of OOE is described to suggest the common acidic components including oxalic acid, malic acid and tartaric acid.
